# Primary metabolism in *Lactobacillus sakei *food isolates by proteomic analysis

**DOI:** 10.1186/1471-2180-10-120

**Published:** 2010-04-22

**Authors:** Anette McLeod, Monique Zagorec, Marie-Christine Champomier-Vergès, Kristine Naterstad, Lars Axelsson

**Affiliations:** 1 Nofima Mat AS, Norwegian Institute of Food, Fisheries and Aquaculture Research, Osloveien 1, NO-1430 Ås, Norway; 2 Department of Chemistry, Biotechnology and Food Science, Norwegian University of Life Sciences, P.O. Box 5003, NO-1432 Ås, Norway; 3 Unité Flore Lactique et Environnement Carné, UR309, INRA, Domaine de Vilvert, F-78350 Jouy en Josas, France

## Abstract

**Background:**

*Lactobacillus sakei *is an important food-associated lactic acid bacterium commonly used as starter culture for industrial meat fermentation, and with great potential as a biopreservative in meat and fish products. Understanding the metabolic mechanisms underlying the growth performance of a strain to be used for food fermentations is important for obtaining high-quality and safe products. Proteomic analysis was used to study the primary metabolism in ten food isolates after growth on glucose and ribose, the main sugars available for *L. sakei *in meat and fish.

**Results:**

Proteins, the expression of which varied depending on the carbon source were identified, such as a ribokinase and a D-ribose pyranase directly involved in ribose catabolism, and enzymes involved in the phosphoketolase and glycolytic pathways. Expression of enzymes involved in pyruvate and glycerol/glycerolipid metabolism were also affected by the change of carbon source. Interestingly, a commercial starter culture and a protective culture strain down-regulated the glycolytic pathway more efficiently than the rest of the strains when grown on ribose. The overall two-dimensional gel electrophoresis (2-DE) protein expression pattern was similar for the different strains, though distinct differences were seen between the two subspecies (*sakei *and *carnosus*), and a variation of about 20% in the number of spots in the 2-DE gels was observed between strains. A strain isolated from fermented fish showed a higher expression of stress related proteins growing on both carbon sources.

**Conclusions:**

It is obvious from the data obtained in this study that the proteomic approach efficiently identifies differentially expressed proteins caused by the change of carbon source. Despite the basic similarity in the strains metabolic routes when they ferment glucose and ribose, there were also interesting differences. From the application point of view, an understanding of regulatory mechanisms, actions of catabolic enzymes and proteins, and preference of carbon source is of great importance.

## Background

*Lactobacillus sakei *is an important food-associated lactic acid bacterium (LAB). Although initially characterized from rice wine [1] and isolated from plant fermentations [2,3] and fermented fish [4,5], its main habitat is meat [6]. It is widely used as starter culture in the production of fermented meat products [7], and is regarded as a potential meat and fish biopreservative [8-10]. *L. sakei *resists harsh conditions which often prevail during preservation, such as high salt concentration, low water activity, low temperature and pH [11]. An important property of the bacterium is the production of lactic acid that acidifies the product and both inhibits growth of spoilage bacteria and food pathogens, and confers taste and texture to the fermented products. The species has also been observed as a transient inhabitant of the human gastrointestinal tract [12-15]. Sequence analysis of the *L. sakei *23K genome has provided valuable information, showing a specialized metabolic repertoire that reflects adaptation to meat products [16].

Among the few sugars available in meat and fish, *L. sakei *utilizes glucose and ribose for growth. The two sugars are fermented through different metabolic pathways: sugar hexose fermentation is homolactic and proceeds via the glycolytic pathway leading to lactate, whereas pentoses are fermented through the heterolactic phosphoketolase pathway ending with lactate and other end products such as acetate [17,18]. A correlation between glucose and ribose metabolism has been suggested for *L. sakei*, and this metabolism could be advantageous in competition with the other microbial flora found on meat [17,19]. With regard to glucose metabolism, the central glycolytic operon, also called the *gap *operon (*cggR-gap-pgk-tpi-eno*), encodes enzymes that catalyse steps of the glycolysis and the putative central glycolytic gene regulator (CggR) [20]. Glucose is transported and phosphorylated by the phosphoenolpyruvate (PEP)-dependent phosphotransferase system (PTS) encoded by the *ptsHI *operon, and by one or more additional non-PTS permeases [18]. A unique *L. sakei rbsUDKR *(LSA0200-0203) gene cluster responsible for ribose catabolism has been described, which encodes a ribose transporter (RbsU), a D-ribose pyranase (RbsD), a ribokinase (RbsK) and the ribose operon transcriptional regulator (RbsR) [16,17,21]. RbsR was shown to function as a local repressor on *rbsUDK*, and as a *ptsI *mutant increased transport and phosphorylation of ribose, the PTS was suggested to negatively control ribose utilization [16,17,21,22]. Moreover, regulation by carbon catabolite repression (CCR) mediated by catabolite control protein A (CcpA) has been suggested, as a putative catabolite responsive element (*cre*) site, the binding site of CcpA, was found preceding *rbsD *[23-25].

It has been proposed that the species can be divided into two subspecies described as *L. sakei *subsp. *sakei *and *L. sakei *subsp. *carnosus *based on results from numerical analyses of total cell soluble protein content and randomly amplified polymorphic DNA (RAPD) patterns [26-28]. *L. sakei *species display a large genomic diversity with more than 25% variation in genome size between isolates [29]. In a previous study, we investigated the diversity of ten *L. sakei *strains by phenotypic and genotypic methods, and could report a wide phenotypic heterogeneity and the presence of two genetic groups which coincide with the subspecies [30]. The growth rates of the strains on glucose and ribose varied, indicating different abilities to metabolize the two sugars. Acidification properties in a meat model also showed differences between the strains, possibly reflecting that some are more suited as starter or protective cultures than others [30]. In this study, we used a proteomic approach to compare the same ten strains, which are isolates from meat and fermented meat products, saké, and fermented fish [30]. We investigated their metabolic routes when growing in a defined medium [31] supplemented with glucose and ribose. Two-dimensional gel electrophoresis (2-DE) combined with mass spectrometry (MS) allowed identification of proteins, the expression of which varied depending on the carbon source used for growth. Previous studies used 2-DE to obtain an overview of global changes in the *L. sakei *proteome as function of uracil deprivation [32], anaerobiosis [33], adaption to cold temperatures and addition of NaCl [34], and high hydrostatic pressure [35]. However, studies on the global protein expression patterns during growth of this bacterium on various carbohydrates have not been reported, and importantly, studies to detect specific differences between strains of *L. sakei *are needed. Our aim in this study was to gain further knowledge about the primary metabolism in *L. sakei*, and to look at strain diversity in this regard.

## Methods

### Bacterial strains, media and growth conditions

The bacterial strains included in this work are listed in Table 1. The organisms were maintained at -80°C in MRS broth [36] (Oxoid) supplemented with 20% glycerol. The complex medium MRS (Oxoid) was used for *L. sakei *propagation, and a completely defined medium (DML) [31], supplemented with either 0.5% glucose (DMLG), 0.5% ribose (DMLR) or 0.5% ribose + 0.02% glucose (DMLRg), was used for liquid cultures. Optical density at 600 nm (OD_600_) was monitored on an Ultrospec 3000 UV/Visible Spectrophotometer (Pharmacia Biotech). Cells were grown at 30°C in MRS to early exponential phase (OD_600 _= 0.2-0.5), before inoculation (about 10^4 ^times diluted) in DML. Under these conditions the cultures were in exponential phase after an overnight incubation. The subcultures were used to inoculate to an initial concentration of 0.07 OD_600 _in fresh DML medium. To monitor the growth rate, flasks containing the cell cultures were stirred moderately to keep bacteria in suspension. For 2-DE analysis samples were prepared from DMLG and DMLRg cultures. Samples were extracted from two independent 100 ml cultures grown to mid-exponential phase (OD_600 _= 0.5-0.6).

**Table 1:** Strains used in this study.

Bacterial strain	Source	Reference
*L. sakei *23K	Sausage	[66,67]
*L. sakei *MF1053	Fermented fish (Norwegian "Rakfisk")	[30]
*L. sakei *LS 25	Commercial starter culture for salami sausage	[68]
*L. sakei *Lb790x	Meat	[69]
*L. sakei *LTH673	Fermented sausage	[70,71]
*L. sakei *MF1328	Fermented sausage	[30]
*L. sakei *MF1058 (TH1)	Vakuum-packed cooked meat, protective culture	[9,10]
*L. sakei *CCUG 31331^a ^(DSM 15831^b^, R 14 b/a)	Fermented sausage, type strain for *L. sakei *subsp. *carnosus*	[27,72]
*L. sakei *DSM 20017^b ^(ATCC 15521^c^)	Sake, alcoholic beverage made by fermenting rice, type strain for *L. sakei *subsp. *Sakei*	[27]
*L. sakei *Lb16 (Lb1048^d^, CCUG 42687^a^)	Minced meat	[31,73]

^a ^CCUG, Culture Collection, University of Gothenburg, Sweden.

^b ^DSM, Deutsche Samlung von Microorganismen und Zellkulturen, Braunschweig, Germany.

^c ^ATCC, American Type Culture Collection, Manassas, VA, USA.

^d ^Designation used in the strain collection at Federal Institute for Meat research, Kulmbach, Germany.

### Extraction of soluble proteins

Proteins were prepared as described by Marceau *et al*. [32] with the following modifications: Cultures of 100 ml were centrifuged at 2800 × *g *at 4°C and washed twice in 0.01 M Tris-HCl buffer, pH 7.5 for 15 min. Bacterial pellets were resuspended in 0.5 ml of the same buffer and 500 mg glass beads were added (acid-washed <106 microns; Sigma-Aldrich). Cells were mechanically disrupted with an FP120 FastPrep cell disruptor (BIO101, Thermo Savant) by four 30 s cycles of homogenization at speed 6.5 with 1 min intervals in ice. Unbroken cells and large cellular debris were removed by centrifugation at 20 800 × *g *for 30 min at 4°C. Protein concentrations of the supernatant (cytosolic fraction) were measured using the colorimetric assay *RC DC *Protein Assay (Bio-Rad), using bovine serum albumin (BSA) as standard protein, according to the manufacturer's instructions. The supernatants were stored in aliquots at -80°C.

### Two-dimensional gel electrophoresis conditions

Aliquots of the *L. sakei *cytosolic fraction corresponding to 50 μg (analytical gel) or 200 μg (preparative gel) of protein were diluted by adding a rehydration buffer (6 M urea (Merck), 2 M thiourea (Merck), 4% 3- [(3-cholamidopropyl)-dimethylammonio]-1-propanesulfonate (CHAPS; Sigma-Aldrich), 0.5% immobilized pH gradient (IPG) buffer pH 4-7 (GE Healthcare Bio-Sciences), and 2.5% dithiothreitol (DTT; Bio-Rad)) to a final volume of 380 μl. This solution was used to rehydrate 18-cm pH 4-7 linear IPG strips (GE Healthcare BioSciences). Strips were passively rehydrated at room temperature for 12-16 h under mineral oil, before isoelectric focusing (IEF) was performed in an Ettan IPGphor II unit (GE Healthcare Bio-Sciences, Uppsala, Sweeden) as follows: 200 V for 1 h, 500 V for 1 h, 1000 V for 1 h, from 1000 to 8000 V in 30 min, and finally 8000 V for 6 h. The strips were incubated at room temperature for 15 min in equilibration buffer (50 mM Tris-HCl pH 8.8, 6 M urea, 30% (v/l) glycerol (Merck) and 2% (w/v) sodium dodecyl sulfate (SDS; Shelton Scientific)) supplemented with 1% (w/v) DTT, followed by 15 min in equilibration buffer containing 2.5% (w/v) iodoacetamide (Merck). SDS-polyacrylamide gel electrophoresis (SDS-PAGE) using 12.5% acrylamide gels was carried out with an Ettan DALT II system (GE Healthcare Bio-Sciences, Uppsala, Sweeden). Proteins were resolved at 20°C at a current of 2.5 mA/gel for 45 min and then at 25 mA/gel until the tracking dye had migrated to the bottom of the gel. Analytical gels were silver stained as described by Blum *et al*. [37] and preparative gels according to Shevchenko *et al*. [38]. For the final analysis, three 2-DE gels were run from each strain from each of the two independent bacterial cultures.

### Image and statistical analysis

Digitized 2-DE images (16-bit greyscale, 300 dpi) of the stained gels were acquired with an office scanner (Epson Perfection 4990 Photo, Epson) and imported into Progenesis SameSpots software v.3.1 (Nonlinear Dynamics). For each strain, five glucose images and five ribose images were aligned using one selected glucose image as a reference [39]. Spots were detected simultaneously across the images leading to one spot map, an approach which addresses the problems of missing values and reduces variance in spot volume across biological or technical replicates by applying the same spot outline across the image series [39,40]. The spot pattern was manually edited, gel artefacts were removed, and images were grouped glucose vs. ribose. An automatic analysis (spot detection, background subtraction, normalisation, and matching) was performed by the software, creating one way ANOVA p-values and q-values as measures of statistical significance, and fold change based on spot normalized volumes of the two groups. Whereas the p-value is a measure of significance in terms of false positive rate, the q-value (or FDR adjusted p-value) is a measure in terms of the false discovery rate (FDR) [41]. Spot normalized volumes were in addition imported into 50-50 MANOVA http://www.langsrud.com/stat/ffmanova.htm for statistical analysis. Rotation tests were performed with 9999 simulations for spot normalized volumes, producing q-values. Differential protein expression was considered to be significant at the level of q < 0.05 from both the SameSpots software and rotation tests, and the expression patterns were checked visually to observe how the spot intensity differed. For strain comparison, a representative image from the sequenced strain *L. sakei *23K was used as a reference. Selected images from each of the other strains from both carbon sources were compared to detect distinct strain differences.

### Protein identification

The protein spots of interest presenting a change in volume depending on carbon source used for growth were excised from preparative gels from the sequenced strain 23K. To confirm the identity of the same spots in other strains, we also excised the spots from strains MF1053 and LS 25. Spots presenting distinct strain differences were excised from strain 23K and MF1053. Samples were prepared for matrix-assisted laser desorption/ionization-time of flight (MALDI-TOF) MS analysis according to the method of Jensen *et al*. [42] with modifications described previously [43]. For purification of digested proteins columns were prepared by packing a plunge of C18 material (3 M Empore C18 extraction disc, Varian) into a gel loader tip (20 μl, Eppendorf). An Ultraflex MALDI-TOF/TOF mass spectrometer with the LIFT module (Bruker Daltonics, GmbH, Bremen, Germany) was used for protein identification. Peptide calibration standard I (Bruker Daltonics) was used for external calibration. The software FlexAnalysis 2.4 (Bruker Daltonics) was used to create peak lists using median baseline subtraction with 0.8 in flatness and smoothing by the Savitzky-Golay filter of 0.2 *m/z *in width. BioTools 3.1 (Bruker Daltonics) was used for interpretation of MS and MS/MS spectra. Proteins were identified by peptide mass fingerprinting (PMF) using the database search program MASCOT http://www.matrixscience.com/, searching against the NCBInr database http://www.ncbi.nih.gov/ with the following settings: Other firmicutes, MS tolerance of 50 ppm and MS/MS tolerance of 0.5 Da, maximum missed cleavage sites was 1, Carbamidomethyl (C) and Oxidation (M) were set as fixed and variable modification, respectively. The number of peptide matches, sequence coverage, pI and MW were used to evaluate the database search results.

## Results and Discussion

In this study, we used proteomics to compare ten *L. sakei *food isolates regarding their metabolic routes when growing on glucose and ribose.

### Growth of *L. sakei *strains on glucose and ribose

The ten strains investigated showed faster growth rates when utilizing glucose as the sole carbon source (DMLG; glucose 0.5%) compared with ribose (DMLR; ribose 0.5%), a finding in agreement with previous observations [16-18,30], confirming that glucose is the preferred carbon source in *L. sakei*. Preliminary 2-DE analysis of strains 23K, MF1053 and LS 25 resulted in gels with large differences in protein spot resolution (results not shown). Gels of samples issued from bacteria grown on ribose as the sole carbon source were of poor quality. Cell proteolysis due to slow growth and prolonged incubation time may result in protein degradation and solubilization defect, as has previously been proposed [44]. Previous studies suggested a regulation of ribose utilization by the PTS and co-metabolism of these two sugars that are present in meat [17,19,21]. Since the addition of small amounts of glucose has been described to enhance growth on ribose [45], we used DMLRg (ribose 0.5%, glucose 0.02%) for further experiments. This indeed resulted in faster growth rates and a better spot resolution of the resulting 2-DE gels that were comparable to the gels from bacterial samples grown in DMLG (results not shown). Thus further experiments were performed by growing bacteria in DMLG and DMLRg to study the glucose and ribose metabolisms, respectively.

### Protein patterns of the ten *L. sakei *strains

**Figure 1:**
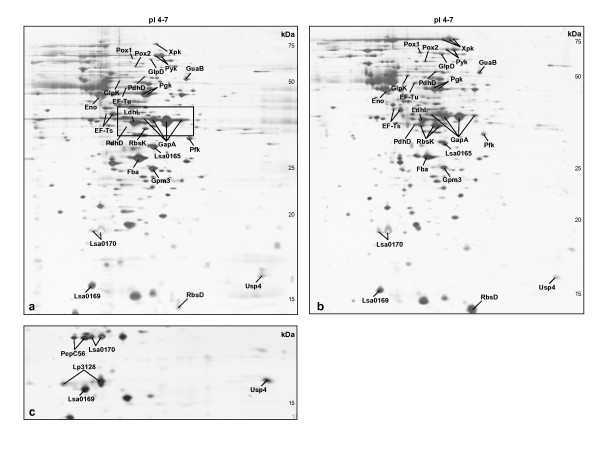
**Silver-stained 2-DE gels images of *Lactobacillus sakei *strain 23K grown in a completely defined medium supplemented with glucose (a) or ribose (b), and the lower part of a 2-DE gel image from *L. sakei *strain MF1053 grown on glucose (c)**. Protein (50 μg) was loaded, and 2-DE was performed using a pH range of 4-7 in the first dimension and SDS-PAGE (12.5%) in the second dimension. Protein size (kDa) is shown on the right side of each gel image. Spots listed in Additional files 1 and 2, Tables S2 and S3 are indicated. The black rectangle (a) shows the region of the GapA isoforms which differ among the strains.

After growth on glucose (in DMLG) and ribose (in DMLRg) an average of approximately 400 spots was observed after 2-DE in the pI range investigated. A variation of about 20% in the number of spots was detected between the strains, as previously observed within the species [29,35]. The overall protein expression pattern was similar for the different strains grown on both carbon sources (data not shown), though distinct differences in the 40-kDa region of the 2-DE gels were observed (Figure 1). These differences were identified as resulting from two different migration profiles of four isoforms (different pI) of the glyceraldehyde-3-phosphate dehydrogenase (GapA) protein. The isoforms displayed a size variation, previously described by Chaillou *et al. *[29] to differentiate two *L. sakei *subgroups. Grouping of our ten strains based on the GapA isoforms migration profile was identical to the two genetic clusters previously obtained from rapidly amplified polymorphic DNA (RAPD), amplified fragment length polymorphism (AFLP), and microarray-based comparative genome hybridization (CGH) analyses [30]. If those grouping methods reflect the subspecies division of *L. sakei*, eight of our strains including the sequenced strain 23K and the type strain CCUG 31331 belong to *L. sakei *subsp. *carnosus*, while the type strain DSM 20017 and the commercial starter culture strain LS 25 belong to *L. sakei *subsp. *sakei*.

Comparison of protein patterns obtained from cells grown on glucose or ribose revealed, for all the strains, differences in the expression profiles. The spots presenting a volume change depending on the carbon source used for growth and identified by MALDI-TOF MS are shown in Figure 1ab in representative 2-DE gel images. All the proteins could be identified against *L. sakei *23K proteins, as shown in Additional file 1, Table S2. Data obtained for a few spots gave less statistically significant results (q = 0.05-0.1) due to co-migration of proteins which made quantification measurements unreliable. However, visual inspection of these protein spots in the 2-DE gels confirmed a modification in their volume. Nine proteins displayed a different level of expression in all tested strains, whereas 11 proteins varied in at least one of the strains (Additional file 1). Moreover, when compared to the other strains we observed that *L. sakei *MF1053 over-expressed a set of seven proteins after growth on both carbon sources, as shown in Additional file 2, Table S3. The proteins could be identified against *L. sakei *23K proteins, except for two proteins which identified against proteins from other *L. sakei *strains and were similar to proteins from *Lactobacillus plantarum *and *Lactobacillus buchneri *(Additional file 2). The presence of several isoforms with different pIs was also noticed for several proteins (Additional files 1 and 2). Many proteins are modified after synthesis by different types of posttranslational modifications (PTM) which may control the protein activity, and the most common PTM accounted for pI differences is phosphorylation [46].

### Proteins differentially expressed between growth on glucose and ribose

**Figure 2:**
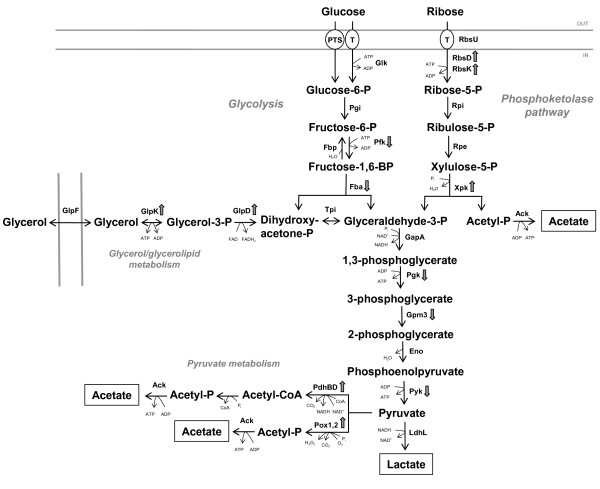
**Overview of the metabolic pathways for glucose and ribose fermentation in *L. sakei***. Enzymes which expression is up- or down-regulated on ribose compared with glucose in the majority of the ten *L. sakei *strains (see Additional file 1, Table S2) are indicated with upward and downward pointing arrows, respectively. End-products are boxed. PTS, phosphotransferase system; T, transport protein; P, phosphate; B, bis; Glk, glucokinase; Pgi, phosphoglucoisomerase; Fbp, fructose-1,6-bisphosphatase; Pfk, 6-phosphofructokinase; Fba, fructose-bisphosphate aldolase; RbsU, ribose transporter; RbsD, D-ribose pyranase; RbsK, ribokinase; Rpi, ribose-5-phosphate isomerase; Rpe, ribulose-phosphate 3-epimerase; Xpk, xylulose-5-phosphate phosphoketolase; Tpi, triose-phosphate isomerase; GapA, glyceraldehyde-3-phosphate dehydrogenase; Pgk, phosphoglycerate kinase; Gpm3, phosphoglycerate mutase; Eno, enolase; Pyk, pyruvate kinase; LdhL, L-lactate dehydrogenase; PdhBD, pyruvate dehydrogenase complex subunits B and D; Pox1,2, pyruvate oxidase; Ack, acetate kinase; GlpD, glycerol-3-phosphate dehydrogenase; GlpK, glycerol kinase; GlpF, glycerol uptake facilitator protein.

In total, ten proteins were up-regulated in all or most of the strains after growth on ribose. Among those, three are directly involved in ribose catabolism: RbsD, the D-ribose pyranase, RbsK, the ribokinase, and Xpk, the putative phosphoketolase. This is in accordance with finding by Stentz *et al*. [17] who observed the induction of the *rbsUDKR *operon transcription and an increase of phosphoketolase and ribokinase activity after growth on ribose. The two pyruvate oxidases and two of the four components of the pyruvate dehydrogenase complex (PDC) were also detected as up-regulated in ribose grow cells. In addition, GlpK and GlpD, the glycerol kinase and glycerol-3-phosphate dehydrogenase were detected in higher quantities in most of the strains after growth on ribose. Conversely, six proteins were down-regulated on glucose, of which four were involved in glycolysis. The inosine-5-monophosphate dehydrogenase (GuaB), involved in purine metabolism, and the putative oxidoreductase Lsa0165 were down-regulated, whereas the elongation factor Ts (EF-Ts) was up-regulated on ribose. An overview of the catabolic pathways for glucose (glycolysis) and ribose (phosphoketolase pathway) utilization in *L. sakei *is shown in Figure 2. Proteins whose expression was modified in cells grown on ribose are shown.

It is likely that the induction of RbsK and Xpk and hence the phosphoketolase pathway in the cells restricts the flow of carbon down the glycolytic route. In many microorganisms, the glycolytic flux depends on the activity of 6-phosphofructokinase (Pfk) and pyruvate kinase (Pyk) [47,48]. Similar to several other LAB [48-50] these two enzymes are encoded from a *pfk-pyk *operon [34], and as reflected at the level of genetic structure, a lower expression of both enzymes was seen on ribose in all strains examined. A lower expression of Pfk was also observed by Stentz *et al*. [17] during growth on ribose. The glycolytic enzymes fructose-1,6-bisphosphate aldolase (Fba) and a phosphoglycerate mutase (Gpm3) showed a lower expression in most of the strains, and interestingly, strains LS 25 and MF1058 showed a lower expression of three more glycolytic enzymes compared to the rest of the strains. It is possible that these strains have a more efficient mechanism of down-regulating the glycolytic pathway. LS 25 is an industrially used starter culture for fermented sausages, while MF1058 is suitable as a protective culture in vacuum packed fresh meat [9,10]. From a meat model system based on minced meat fermentation we previously observed that these two strains performed the fastest acidification of the ten strains, and also had the ability to compete with the indigenous microbiota of the meat batter [30]. Although the triose-phosphate isomerase (Tpi), GapA, phosphoglycerate kinase (Pgk), and enolase (Eno) are all encoded from the *gap *operon [20], our proteome data showed a significantly lower expression only for GapA, Pgk and Eno. In addition, expression of the L-lactate dehydrogenase (LdhL) responsible for the reduction of pyruvate to lactic acid was observed to be lower in the two strains.

The bacterium alters its pyruvate metabolism growing on ribose compared to glucose, possibly since during ribose utilization, more ATP is generated from pyruvate per ribose unit when acetate is produced than when lactate is produced [51]. The up-regulated pyruvate oxidases convert pyruvate into acetyl-phosphate, and the PDC catalyses the transformation of pyruvate to acetyl-CoA (Figure 2).

The increased GlpD enzyme belongs to the glycerol/glycerolipid catabolic pathway, a pathway linked to membrane properties as glycerol-3-phosphate can be converted to phosphatidic acid, which leads to membrane phospholipid synthesis. Also when exposed to low temperature, this protein shows an increased expression in *L. sakei *[34]. Modified membrane properties could potentially also exist as a response to the higher level of acetate produced when utilizing ribose. Acetate has a higher antimicrobial effect than lactate, with pK_a _values of 4.74 and 3.86, respectively, and the proportion of antimicrobial undissociated acetic acid molecules is increased as the pH is lowered. The *glpD *gene is associated in a *glp *operon with glycerol kinase (*glpK*), which also showed an increased expression on ribose, and glycerol uptake facilitator protein (*glpF*) genes [34].

The role of CcpA in CCR in *L. plantarum *has previously been established, and CcpA was shown to mediate regulation of the *pox *genes encoding pyruvate oxidases [52,53]. Rud [54] observed an up-regulation of several genes and operons including the *pox *genes, the *pdh *operon encoding the PDC, and the *glp *operon, during growth on ribose compared with glucose. As putative *cre *sites [55] were identified in promoter regions, their expression was suggested to be regulated by CcpA-mediated CCR. The putative *cre *site found preceding *rbs *in *L. sakei *[25], could indicate that this bacterium possesses global regulation mediated by CcpA. In an *rbsR *mutant overexpressing RbsUDK, the growth on ribose was not accelerated, whereas in a *ptsI *mutant, the transcription of *rbsUDK *was not modified, but transport and phosphorylation of ribose increased. Thus it was concluded that the PTS negatively controls ribose utilization, by a direct or indirect way [17,22]. Nevertheless, a change in expression of the PTS enzymes could not be detected in our ribose 2-DE gels. Further experiments are needed to elucidate the mechanism by which the *rbs *operon is regulated.

The EF-Ts, with an increased expression on ribose, is involved in protein synthesis and translation elongation, and the less expressed GuaB is involved in nucleotide biosynthesis, where ribose is a source for the basic molecule phosphoribosylpyrophosphate (PRPP). Finally, the putative oxidoreductase Lsa0165, also less expressed on ribose, belongs to the short-chain dehydrogenases/reductases family (SDR), possibly a glucose dehydrogenase.

### Proteins over-expressed in *L. sakei *MF1053

Interestingly, compared to the other strains *L. sakei *MF1053 showed a higher expression of seven proteins related to stress whatever the carbon source used for growth (Figure 1c). A list of the proteins and references where their involvement in different stresses are described [56-65], are listed in Additional file 2, Table S3. The reason for the observed difference in expression of these stress proteins remains to be elucidated.

## Conclusions

At present, the complete *L. sakei *genome sequence of strain23K is available [16], and the genome sequence of strain DSM 15831 is currently under assembly http://www.ncbi.nlm.nih.gov/genomes/lproks.cgi. It is obvious from the data obtained in this study that the proteomic approach efficiently identify differentially expressed proteins caused by the change of carbon source. However, the absence of genome sequence remains a limiting factor for the identification of proteins in the non sequenced strains. Sequence analysis has provided valuable information, showing a metabolic repertoire that reflects adaptation to meat, though genomic analyses provide a static view of an organism, whereas proteomic analysis allows a more dynamic observation. Despite the basic similarity in the strains metabolic routes when they ferment glucose and ribose, there were also differences. We are currently combining proteomic and transcriptomic data of different *L. sakei *strains and hope to reveal more about the primary metabolism. From the application point of view, to understand regulatory mechanisms, actions of catabolic enzymes and proteins, and preference of carbon source is of great importance.

## Abbreviations

2-DE: two dimensional gel electrophoresis; MALDI-TOF MS: matrix-assisted laser desorption/ionization-time of flight mass spectrometry; MW: molecular weight; pI: isoelectric point; RbsD: D-ribose pyranase; RbsK: ribokinase; Fbp: fructose-1,6-bisphosphatase; Pfk: 6-phosphofructokinase; Fba: fructose-bisphosphate aldolase; Xpk: xylulose-5-phosphate phosphoketolase; Tpi: triose-phosphate isomerase; GapA: glyceraldehyde-3-phosphate dehydrogenase; Pgk: phosphoglycerate kinase; Gpm: phosphoglycerate mutase; Eno: enolase; Pyk: pyruvate kinase; LdhL: L-lactate dehydrogenase; PDC: pyruvate dehydrogenase complex; Pox: pyruvate oxidase; Ack: acetate kinase; GlpD: glycerol-3-phosphate dehydrogenase; GlpK: glycerol kinase; GuaB: inosine-5-monophosphate dehydrogenase; EFTs: elongation factor Ts; CCR: carbon catabolite repression; CcpA: catabolite control protein A.

## Authors' contributions

AM participated in the design of the study, conducted the experimental work, image and statistical analysis, analyzed and interpreted data, and drafted the manuscript. MZ, MCCV, KN and LA conceived the study, participated in the study design process, and helped write the manuscript. All authors read and approved the final manuscript.

## Supplementary Material

Additional file 1 **Table S2. Identification of protein spots differentially expressed depending on the carbon source used for growth in ten *L. sakei *strains**. Presents identification and characteristics of protein spots with a significant volume change depending on the carbon source used for growth in ten *L. sakei *strains. Click here for file

Additional file 2 **Table S3. Proteins over-expressed in *L. sakei *MF1053**. Presents the identification and characteristics of protein spots over-expressed in *L. sakei *MF1053 compared to the other *L. sakei *strains in this study. Click here for file
