# Supplementary material for: Primary metabolism in Lactobacillus sakei food isolates by proteomic analysis
Source: BMC Microbiol. 2010 Apr 22;10:120. doi: 10.1186/1471-2180-10-120 (PMC2873491; doi:10.1186/1471-2180-10-120)
Supplement: Additional file 1 — Table S2. Identification of protein spots differentially expressed depending on the carbon source used for growth in ten L. sakei strains. Presents identification and characteristics of protein spots with a significant volume change depending on the carbon source used for growth in ten L. sakei strains. [file 1471-2180-10-120-S1.PDF]

Table S2. Protein spots with a volume change depending on the carbon source used for growth in ten *L. sakei* strains. The proteins were identified against *L. sakei* 23K (Lsa) proteins. "+" and "-" refer to a statistically significant ($q < 0.05$) higher and lower expression on ribose compared with glucose, respectively.

Functional category	Spot name[a]	Protein identification	NCBI GI identifier	aa	Matched peptides / % sequence coverage[b]	MW[c] / MW[d] (x 10^3)	pI[c] / pI[d]	Average protein level ratio (fold change)[e, f]									
								23K	MF 1053	LS 25	Lb 790x	LTH 673	MF 1328	MF 1058	CCUG 31331	DSM 20017	Lb 16
**Carbohydrate metabolism**																	
Phosphoketolase pathway	RbsK	Ribokinase Lsa0202	gi|81427812	302	18 / 74	31.66 / 34	5.01 / 4.9, 5.0, 5.05	++	++	++	++	++	++	++	++	++	++
	Xpk	Putative phosphoketolase Lsa0289	gi|81427904	787	19 / 21	88.70 / 86	5.14 / 5.10, 5.15, 5.2	++	++	++	+++	++	+++	++	++	++	++
Pyruvate metabolism	Pox1	Pyruvate oxidase Lsa1188	gi|81428800	611	31 / 66	66.76 / 66	4.97 / 4.9	+	++	++	++	++	++	++	++	++	+
	Pox2	Pyruvate oxidase Lsa1830	gi|81429441	577	17 / 30	62.36 / 62	4.99 / 4.95	+	no	no	+	no	+	+	+	no	no
	PdhB	Pyruvate dehydrogenase complex E1-β subunit Lsa1084	gi|81428694	332	10 / 41	36.13 / 36	5.09 / 4.8	++	++	++	++	++	++	++	++	++	++
	PdhD	Dihydrolipoamide dehydrogenase Lsa1082	gi|81428692	468	10 / 24	49.64 / 52	5.07 / 5.0	+	++	+	++	+[g]	+	+[g]	++	+	+
Glycolysis	Fba	Fructose-bisphosphate aldolase Lsa1527	gi|81429140	287	11 / 46	30.88 / 28	4.93 / 4.95	-	-	-[g]	no	-	-	- -	-	-[g]	no
	Pyk	Pyruvate kinase Lsa1032	gi|81428643	586	31 / 66	62.92 / 68	5.19 / 5.15, 5.2	-	- -	-	-	- -	-[g]	- -	-	-[g]	-
	Gpm3	Phosphoglycerate mutase Lsa0206	gi|81427816	229	9 / 54	25.85 / 25	5.04 / 5.1	-	-	-	-[g]	-	no	- -	-[g]	-	-
	Pfk	6-phosphofructokinase Lsa1033	gi|81428644	319	24 / 70	34.34 / 32	5.36 / 5.4	-	-	-	-[g]	-	-[g]	-	-	-	-
	Pgk	Phosphoglycerate kinase Lsa0605	gi|81428220	404	20 / 62	42.72 / 42	5.03 / 5.0, 5.05	no	no	-	no	no	no	- -	no	no	no
	GapA	Glyceraldehyde-3-phosphate dehydrogenase Lsa0604	gi|81428219	338	16 / 53	35.47 / 35	5.19 / 5.0, 5.1, 5.2, 5.3	no	no	-	no	no	no	-	no	no	no
	LdhL	L-lactate dehydrogenase Lsa1606	gi|81429218	325	28 /82	35.42 / 34	4.99 / 4.9	no	no	-	no	no	no	-	no	no	no
	Eno	Phosphopyruvate hydratase / enolase Lsa0607	gi|81428222	431	28 /66	46.55 / 41	4.70 / 4.6	no	no	-	no	no	no	-	no	no	no
**Membrane transport**	RbsD	D-ribose pyranase Lsa0201	gi|81427811	131	5 / 52	14.30 / 14	5.32 / 5.3	+++	+++	+++	++	++	+++	++	++	+++	++
**Glycerol/glycerolipid metabolism**	GlpD	Glycerol-3-phosphate dehydrogenase Lsa0650	gi|81428265	608	44 / 74	66.77 / 66	5.13 / 5.05	++	+++	++	++	++	+++	+++	+++	++	++
	GlpK	Glycerol kinase Lsa0276	gi|81428263	505	22 / 39	55.89 / 51	4,89 / 4.75	++	++	+	no	++	no	+	+	no	++
**Nucleotide metabolism**	GuaB	Inositol-5-monophosphate dehydrogenase Lsa0276	gi|81427891	493	28 / 68	52.30 / 52	5.39 / 5.35	no	-	-	no	-	-	no	no	-	-
**Oxidoreductase activity**	Lsa0165	Putative oxidoreductase Lsa0165	gi|81427775	296	15 / 60	31.33 / 31	5.09 / 5.1	no	-	-	no	- -	-	-	no	no	-
**Protein synthesis**	EF-Ts	Elongation factor Ts Lsa1264	gi|81428875	291	15 / 51	31.31 / 38	4.82 / 4.65, 4.7	+	no	no	+	++	no	++	+	no	+

[a] Spot name referring to the spots labelled in Figure 1.

[b] Matched peptides and percentage of amino acid coverage for the 23K strain.

[c] Theoretical MW (kDa) and pI values.

[d] Estimated MW (kDa) and pI values by 2-DE from strain 23K. Several pI values for one protein refer to different isoforms.

[e] Average fold change increase (+) or decrease (-) on ribose gels compared to glucose gels. +++, > 5; ++, 2-5; +, 1.5-2; -, 1.5-2; - -, 2-5.

[f] no, no change in expression.

[g] $0.05 > q > 0.1$.
