# Supplementary material for: Primary metabolism in Lactobacillus sakei food isolates by proteomic analysis
Source: BMC Microbiol. 2010 Apr 22;10:120. doi: 10.1186/1471-2180-10-120 (PMC2873491; doi:10.1186/1471-2180-10-120)
Supplement: Additional file 2 — Table S3. Proteins over-expressed in L. sakei MF1053. Presents the identification and characteristics of protein spots over-expressed in L. sakei MF1053 compared to the other L. sakei strains in this study. [file 1471-2180-10-120-S2.PDF]

Table S3. Protein spots over-expressed in *L. sakei* MF1053 compared to the other *L. sakei* strains in this study. "+" refers to a statistically significant ($q < 0.05$) higher expression on 2-DE gels from growth in both DMLG and DMLRg compared to the reference *L. sakei* 23K.

Spot name[a]	Protein identification[b]	NCBI GI identifier	aa	Matched peptides / % sequence coverage	MW[c] / MW[d] (x 10^3)	pI[c]/pI[d]	Average protein level ratio (fold change)[e, f]		Protein function, stress, reference
							23K	MF1053	
GuaB	Inositol-5-monophosphate dehydrogenase Lsa0276	gi|81427891	493	28 / 68[g]	52.30 / 52[g]	5.39 / 5.35[g]	p	++	Nucleotide biosynthesis. Superoxide stress in *Bacillus subtilis* [56] and salt stress in *Listeria monocytogenes* [57,58].
EF-Tu	Elongation factor Tu Lsa1063	gi|81428673	396	15 / 40[g]	43.27 / 43[g]	4.72 / 4.8[g]	p	+++	Translation elongation, protein folding and protection from stress. High-pressure in *Lactobacillus sanfranciscensis* [59] and salt stress in *Listeria monocytogenes* [65,66].
Lsa0169	Putative general stress protein Lsa0169	gi|81427779	142	7 / 38[g]	15.62 / 15[g]	4.71 / 4.55[g]	p	+	General stress. High-pressure in *L. sakei* [35]. Related to alkaline shock proteins (Asp) in *L. plantarum* [60].
Lsa0170	Putative general stress protein Lsa0170	gi|81427780	153	13 / 69[g]	17.06 / 17[g]	4.68 / 4.55, 4.65[g]	p	++	General stress. High-pressure [35] and at cold temperature in *L. sakei* [34]. Related to alkaline shock proteins (Asp) in *L. plantarum* [60].
Usp4	Universal stress protein Lsa1173	gi|81428783	144	8 / 65[g]	15.64 / 16[g]	5.93 / 5.9[g]	p	+++	Universal stress UspA protein family. Six Usp paralogs are present in the *L. sakei* 23K genome [16]. High-pressure in *L. sakei* [35].
Lp3128	Stress induced DNA binding protein from *L. plantarum* WCFS1	giI28379536	155	9 / 47[h]	18.01 / 16[h]	4.70 / 4.35, 4.7[h]	np	++	DNA protection during starvation (Dps) protein family. Oxidative, acid, osmotic, high-pressure stresses in *E. coli* [61-64].
	Putative ferritin-like DNA binding protein from *L. sakei*	gi|189085575	155	9 / 47[h]	18.04 / 16[h]	4.70 / 4.35, 4.7[h]			
PepC56	C56 family peptidase from *L. sakei* DSM 15831 (genome sequencing status 'assembly')	gi|227361883	167	8 / 55[h]	18.16 / 17[h]	4.47 / 4.45, 4.5[h]	np	+++	Degradation of aberrant and non-functional proteins [65].
	C56 family peptidase from *L. buchneri* ATCC 11577	giI227513849	167	8 / 55[h]	18.36 / 17[h]	4.65 / 4.45, 4.5[h]			

[a] Spot name referring to the spots labelled in Figure 1.
[b] Lsa refers to *L. sakei* 23K.
[c] Theoretical MW (kDa) and pI values.
[d] Estimated MW (kDa) and pI values by 2-DE. Several pI values for one protein refer to different isoforms.
[e] p, spot present; np, spot not present.
[f] Average fold change increase (+) on glucose gels using strain 23K as a reference. +++, > 5; ++, 2-5; +, 1-2.
[g] Estimated MW (kDa) and pI values, and matched peptides and percentage of amino acid coverage are shown from strain 23K.
[h] Estimated MW (kDa) and pI values, and matched peptides and percentage of amino acid coverage are shown from strain MF1053.
